# Microbial Role in Straw Organic Matter Depolymerization to Dissolved Organic Nitrogen Under Nitrogen Fertilizer Reduction in Coastal Saline Paddy Soil

**DOI:** 10.3390/microorganisms13102333

**Published:** 2025-10-10

**Authors:** Xianglin Dai, Jianping Sun, Hao Li, Zijing Zhao, Ruiping Ma, Yahui Liu, Nan Shan, Yutao Yao, Zhizhong Xue

**Affiliations:** 1 Institute of Coastal Agriculture, Hebei Academy of Agriculture and Forestry Sciences, Tangshan 063200, China; bhssjp@163.com (J.S.); nkybhslh@126.com (H.L.); zhaozijing94@163.com (Z.Z.); marp0825@126.com (R.M.); yutao890310@163.com (Y.Y.); nvtw_306675@sohu.com (Z.X.); 2 Tangshan Key Laboratory of Rice Breeding, Tangshan 063200, China; 3 National Center of Technology Innovation for Comprehensive Utilization of Saline-Alkali Land, Dongying 257300, China; 4 School of New Materials and Chemical Engineering, Tangshan University, Tangshan 063000, China; shannan@tsc.edu.cn

**Keywords:** alkaline protein-degrading bacteria, chitin-degrading bacteria, *apr* and *chiA* genes, nitrogen hydrolase, nitrogen depolymerization pathway

## Abstract

This study examines the effects of reduced nitrogen (N) application on rice straw N depolymerization in coastal saline paddy soil to establish a scientific basis for optimizing N application strategies during straw incorporation in coastal paddy systems. A 360-day field straw bag burial experiment was conducted using four N application levels: N0 (control, without N fertilizer), N1 (225 kg N/ha), N2 (300 kg N/ha), and N3 (375 kg N/ha). The results indicated that applying 300 kg N/ha significantly (*p* < 0.05) increased dissolved organic N (DON) content, *apr* and *chiA* gene copies, and the activities of alkaline protease, chitinase, leucine aminopeptidase, and N-acetylglucosaminidase. In addition, the application of 300 kg N/ha enhanced the synergistic effects of alkaline protein- and chitin-degrading microbial communities. *Pseudomonas*, *Brevundimonas*, *Sorangium*, *Cohnella*, and *Thermosporothrix* were identified as keystone taxa predominant in straw N depolymerization. Straw N depolymerization occurred by two primary pathways: direct regulation of enzyme activity by straw properties of total carbon and electrical conductivity, and indirect influence on N hydrolase activity and DON production through modified microbial community structures. The findings suggest that an application rate of 300 kg N/ha is optimal for promoting straw N depolymerization in coastal saline paddy fields.

## 1. Introduction

Coastal saline–alkali lands, covering over one million hectares in China, are crucial arable reserves essential for national food security [1]. Rice cultivation effectively reclaims these soils through its salt-suppressing properties. However, excessive nitrogen (N) fertilization has resulted in low N use efficiency—averaging only 33.3% [2], significantly below the 2025 national target of 43%, leading to environmental challenges including soil degradation and eutrophication [3]. Therefore, developing optimized N fertilizer management strategies that maintain both productivity and sustainability in these regions is an urgent agricultural priority [4].

Crop straw, a primary byproduct of agricultural production, contains abundant N, phosphorus (P), potassium (K), and trace elements. N released during straw decomposition can supplement synthetic N fertilizer, enhance N availability for crops, and increase soil N storage capacity. Multiple studies indicate that reducing N fertilizer inputs combined with straw returning effectively achieves N fertilizer substitution and improves N use efficiency in agricultural systems [5,6]. However, research has also shown that the inherently high carbon-to-N (C/N) ratio of straw promotes microbial immobilization of soil inorganic N (IN; NH_4_^+^-N and NO_3_^−^-N) to meet metabolic demands during decomposition. This process creates competition between soil microbes and crops, potentially reducing crop yields [7]. Therefore, enhancing straw N depolymerization and subsequent mineralization to IN under reduced N fertilizer application remains critical to addressing these challenges.

Straw N initially undergoes depolymerization to dissolved organic N (DON) through microbial activity before conversion to IN [8]. The depolymerization of straw N to DON is the initial and rate-limiting step of this process. Recent studies have identified protease and chitinase (CAS) as reliable indicators of organic N depolymerization potential, reflecting overall microbial metabolic capacity [9,10,11]. The functional genes *npr* and *apr* play crucial roles in N depolymerization, encoding neutral protease and alkaline protease (AP) enzymes, respectively [12,13]. These genes serve as effective molecular markers for identifying microbial taxa potentially involved in straw N depolymerization [14,15,16]. Studies using *apr* gene sequencing have revealed dominant microbial taxa responsible for protease-mediated hydrolysis and enhanced N depolymerization in corn rhizosphere soils, including the genera *Pseudomonas*, *Caulobacter*, *Yersinia*, and *Labrenzia* [17]. Similarly, based on chiA sequencing, Lu et al. [18] identified *Stenotrophomonas*, *Streptomyces*, *Actinoplanes*, and *Lysobacter* as keystone genera in alpine meadow soils of the Qinghai–Tibetan plateau under four restoration regimes. In addition, leucine aminopeptidase (LAP) and N-acetylaminoglucosidase (NAG) serve as sensitive indicators of N depolymerization, providing valuable insights into key biological N-cycling processes [19]. Most studies focus on soil organic N mineralization, while relatively few address straw N depolymerization. Specific microbial communities may be recruited in response to changes in straw properties, including carbon (C) content, N source concentration or forms, electrical conductivity (EC), and pH, as well as external factors such as N application rate and temperature at different decomposition stages. Thus, directly profiling straw-colonizing microorganisms provides a targeted approach to identifying key taxa involved in regulating straw N depolymerization.

Soil microorganisms operate within complex interspecies networks that fundamentally regulate community structures and ecosystem processes [20]. Molecular ecological network analysis has recently been widely adopted for its ability to visualize intricate microbial interactions and their responses to environmental factors [21]. Furthermore, interactions among microbial communities determine their functioning, with keystone taxa often exerting disproportionately greater influence than other co-occurring communities [22,23]. Fertilization and straw returning significantly shape microbial co-occurrence networks [24,25]. Microorganisms may occupy functionally distinct ecological niches and trophic groups, enabling coexistence based on specific nutritional preferences [17]. For instance, reduced N application can alleviate competitive pressures among bacterial species, promoting a more diverse and stable microbial ecosystem [25]. However, changes in the relationship between straw N-depolymerizing microorganism co-occurrence networks following N application rate remain understudied. Understanding this mechanism is crucial for revealing how N application alters straw N depolymerization through microbial interactions and network dynamics, and is essential for optimizing fertilization strategies by straw returning in coastal paddy systems.

The objectives of this study were to (i) clarify the effects of N fertilizer reduction on straw properties, N hydrolase activities, and N-depolymerizing microbial community structure; (ii) identify keystone taxa driving straw N depolymerization in coastal saline paddy soil; and (iii) elucidate the regulatory pathways of straw N depolymerization.

## 2. Materials and Methods

### 2.1. Site Description

The study was conducted at the Coastal Agricultural Comprehensive Experimental Station of the Hebei Academy of Agriculture and Forestry Sciences, Caofeidian District, Hebei Province, China (39°17′ N, 118°27′ E). This area within the Bohai Rim lies in a warm temperate zone and experiences a semi-humid monsoon climate characterized by an average annual temperature of 10.8 °C, annual precipitation of 636 mm, and a frost-free period of approximately 188 days. The experimental site contains saline paddy soil, classified as Salic Anthrosol according to the World Reference Base for Soil Resources [26]. The site maintains a rice monoculture system. Table 1 presents the initial soil properties (0–20 cm depth) recorded before the experiment. Rice straw used in the experiment was harvested at maturity in 2022, and its characteristics are detailed in Table 1.

### 2.2. Experimental Design and Soil Sampling

**Figure 1:**
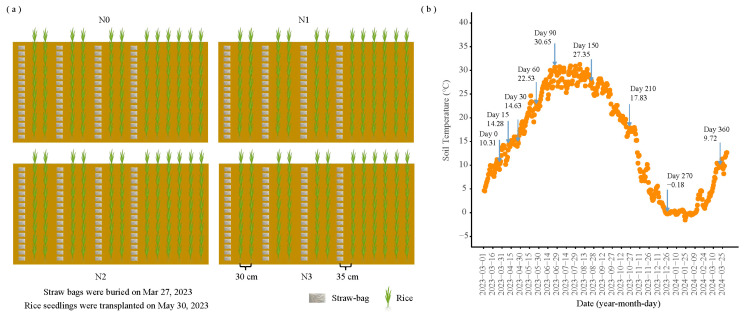
(**a**) Schematic representation of straw-bag positioning. (**b**) Soil temperature measured at 20 cm depth. Numbers above the arrow indicate the duration of straw-bag burial in days and the corresponding soil temperature. N0 (control, without N fertilizer), N1 (225 kg N/ha), N2 (300 kg N/ha), and N3 (375 kg N/ha).

A straw decomposition burial experiment commenced in 2023 with four N application treatments: N0 (control, without N fertilizer), N1 (225 kg N/ha; 40% reduction in conventional N application), N2 (300 kg N/ha; 20% reduction in conventional N application), and N3 (375 kg N/ha; conventional N application). The burial experiment was performed with 64 straw bags per treatment using the nylon mesh bag method (bag dimensions: 15 × 10 cm; mesh aperture: 50 μm). Rice straw was oven-dried at 60 °C and cut into 3–5 cm segments before burial. Prior to the experiment, the topsoil was thoroughly homogenized and divided into four separate plots (to prevent cross-flow of water and fertilizers). Each plot corresponds to a straw bale under different nitrogen application treatments. Each nitrogen application treatment comprised 64 straw bales, and eight bales were randomly selected per sampling, with a total of eight samplings (8 times × 8 replicates). Each bag contained 17.0 g of straw, equivalent to a local field application rate of 10,500 kg/ha. Urea was applied at rates of 0 (N0, control), 0.79 (N1), 1.07 (N2), and 1.33 (N3) g per bag, corresponding to the field N application levels. After thorough mixing, the straw was sealed in the nylon mesh bags and buried horizontally at a depth of 20 cm. The experimental layout is illustrated in Figure 1a. Temperature dynamics were monitored using a ground thermometer installed at 20 cm depth (Figure 1b). Straw bags were buried on 27 March 2023, with sampling conducted at 15, 30, 60, 90, 150, 210, 270, and 360 days post-burial, retrieving eight bags per treatment at each time point. Samples were transported under cold-chain conditions to the laboratory for processing. Fresh straw was divided into three portions for various analyses. The first portion was oven-dried at 60 °C, milled, and sieved through 0.5 mm mesh for determination of total C (TC), total N (TN), total P (TP), total K (TK), pH, and EC; the second portion was refrigerated at 4 °C for analysis of DON and N hydrolase activities; and the third portion was stored at −80 °C for microbial community structure analysis.

### 2.3. Measurements and Methods

#### 2.3.1. Analysis of Straw Properties

The straw samples underwent drying to constant weight, grinding, and sieving through a 0.5 mm mesh. TC and TN contents were analyzed using an elemental analyzer (Elementar Analysensysteme GmbH, Hanau, Germany). For TP and TK analyses, 0.2 g of the sample (accurate to 0.0001 g) underwent digestion with peroxymonosulfuric acid. TP was quantified using vanadate–molybdate–yellow colorimetry, while TK was measured by flame photometry. DON determination used the differential subtraction method. Fresh straw (10 g) was combined with 50 mL of 2 mol/L potassium chloride solution in a 100 mL centrifuge tube, maintaining a 1:5 straw-to-water ratio. The mixture underwent shaking at 250 rpm for 1 h, followed by 30 min of settling, and filtration through a 0.45 μm membrane. Total dissolved N (TDN) and IN (NH_4_^+^-N and NO_3_^−^-N) concentrations in the filtrate were measured using an elemental analyzer (Elementar Analysensysteme GmbH) and a continuous flow injection analyzer (FLAstar 5000 Analyzer; Foss Tecator, Hillerød, Denmark), respectively. DON was calculated as the difference between TDN and IN. For pH and EC analyses, dried straw (1 g, particle size < 0.5 mm) was combined with 20 mL of deionized water at a 1:20 (*w*/*v*) ratio. Following 90 min of shaking and 30 min of settling, pH was measured using a pH meter, and EC was determined using a conductivity meter (DDSJ-308F; Shanghai Yidian Scientific Instrument Co., Ltd., Shanghai, China).

#### 2.3.2. Straw N Hydrolase Activity Analysis

NAG and LAP activities were determined using a microplate fluorometric protocol [3]. Specifically, 1.0 g of fresh straw was homogenized in 100 mL of sterilized water using a polytron homogenizer, followed by continuous stirring with a magnetic stirrer to ensure uniform suspension. Sterilized water, the homogenized sample, 10 μM reference standards, and 200 μM substrates were added to wells of a black 96-well microplate and incubated in darkness at 25 °C for 4 h. The reactions were terminated by adding 10 μL of 1.0 M sodium hydroxide. Fluorescence measurements were then taken at an excitation wavelength of 365 nm and an emission wavelength of 450 nm using a multi-mode microplate reader (Fluoroskan Ascent FL; Thermo Fisher Scientific, Waltham, MA, USA). Enzymatic activities are expressed as nmol/g/h.

Straw AP and CAS activities were determined according to protocols provided with the respective assay kits (Solarbio Science and Technology Co., Ltd., Beijing, China). The AP assay relied on the enzymatic hydrolysis of casein under alkaline conditions, releasing tyrosine, which reduces phosphomolybdic acid to form tungsten blue, a chromogen with maximum absorbance at 680 nm. AP activity was quantified based on the rate of absorbance increase at 680 nm and expressed as one unit of straw AP activity, defined as the amount of enzyme producing 1 μmol of tyrosine per gram of straw per day (μmol/g/d). For CAS activity, chitin hydrolysis releases N-acetylglucosamine, which undergoes alkali-catalyzed conversion to an intermediate that reacts with p-dimethylaminobenzaldehyde, forming a chromophore with peak absorption at 585 nm. CAS activity was calculated from the absorbance increase at 585 nm and expressed as one unit of enzyme activity, defined as the amount required to decompose chitin and produce 1 μg of N-acetylglucosamine per gram of straw per day (μg/g/d).

#### 2.3.3. DNA Extraction and Real-Time Quantitative Polymerase Chain Reaction (qPCR)

Genomic DNA was extracted from 0.5 g of fresh straw using the E.Z.N.A.^®^ Soil DNA Kit (Omega Bio-Tek, Norcross, GA, USA). DNA concentration and purity were evaluated using 1% (*w*/*v*) agarose gel electrophoresis and a NanoDrop 2000 spectrophotometer (Thermo Fisher Scientific). qPCR amplification of the *apr* and *chiA* genes was conducted on an ABI 7500 system (Applied Biosystems, Foster City, CA, USA) using the primer pairs FaprI/RaprI (5′-TAYGGBTTCAAYTCCAAYAC-3′/5′-VGCGATSGAMACRTTRCC-3′ [12] and chif1/chif2 (5′-ATCTTCGCTGGGTCGGCTGG-3′/5′-GACGGCATCGACATCGATTGG-3′ [16], respectively. The GenBank accession numbers for the target genes for *apr* and *chiA* used for calibration were AB013895 and AJ812562, respectively. The amplification protocol for the *apr* gene comprised pre-denaturation at 95 °C for 3 min, followed by 40 cycles of denaturation at 95 °C for 15 s, annealing at 52 °C for 20 s, and extension at 72 °C for 20 s. Each sample was analyzed in triplicate. A 30 μL PCR system (Q321-02/03; Vazyme Biotech Co., Ltd., Nanjing, China) was used, containing 15 μL of 2× Mix, 0.5 μL each of forward and reverse primers (10 μM), 2 μL of DNA template, and double-distilled water added to reach 30 μL final volume. The standard curve for the *apr* gene yielded an *R*^2^ value of 0.995 and an amplification efficiency of 84.2%. The amplification protocol for the *chiA* gene involved pre-denaturation at 95 °C for 3 min, followed by 40 cycles of denaturation at 95 °C for 15 s, annealing at 57 °C for 20 s, and extension at 72 °C for 20 s. Each reaction was performed in triplicate. The same 30 μL PCR system (Q321-02/03) was used. The standard curve for the chiA gene demonstrated an *R*^2^ value of 0.995 with an amplification efficiency of 81.9%.

#### 2.3.4. High-Throughput Sequencing and Bioinformatics Analyses

PCR products were quantified using Qubit^®^ 3.0 (Life Invitrogen, Carlsbad, CA, USA). Sets of 24 amplicons with unique barcodes were combined in equal proportions. A DNA library was constructed by unwinding the DNA duplex product after PCR amplification and removing uncircularized DNA molecules. Libraries were sequenced on a next-generation sequencing platform (Shanghai Biozeron Biotech Co., Ltd., Shanghai, China) using paired-end 300 bp reads, following standard protocols. The raw sequence data have been deposited in the Genome Sequence Archive at the National Genomics Data Center, China National Center for Bioinformation/Beijing Institute of Genomics, Chinese Academy of Sciences (GSA: CRA027124 and CRA027210), accessible at https://ngdc.cncb.ac.cn/gsa (accessed on 23 June 2025). The bioinformatics analyses and data processing methods are detailed in the Supplementary Materials.

### 2.4. Statistical Analysis

Data were organized using Microsoft Excel (v2019; Microsoft Corp., Redmond, WA, USA). All experiments were performed with at least four replicates (*n* = 4, experimental replication). Statistical analyses were performed using R software (v4.5.0). One-way analysis of variance (ANOVA) with Duncan’s post hoc test (α = 0.05) was used to identify significant differences among fertilizer treatments or decomposition stages. Spearman correlations between *apr* and *chiA* gene levels, straw properties, and N hydrolase enzyme activity were calculated using the “corrplot” package (version 0.84). Correlation heatmaps were generated using the “heatmap3” package (version 1.1.9). Unconstrained principal coordinate analysis (PCoA) based on unweighted UniFrac distances was performed to evaluate bacterial community structure. The effects of N application and decomposition duration on bacterial community structure were assessed using permutational multivariate analysis of variance (PERMANOVA) with 999 permutations, implemented through the adonis function in the “vegan” package (version 2.5.7). The “rdaccaa.hp” package (version 1.1-1) quantified the contributions of environmental factors to microbial community structures [27]. The “randomForest” package (version 4.6.14) was used to construct a random forest model evaluating the relative importance of predictor variables based on mean decrease accuracy (MDA). Higher MDA values indicate stronger predictive power and associative strength for response variables, thereby identifying key predictors for biomarker selection [28]. The structural associations among N application, temperature, straw properties, N hydrolase enzyme activity, and microbial community structure were analyzed using partial least squares path modeling (PLS-PM) by the “pls” package (version 0.4.9).

Network analysis investigated co-occurrence patterns of alkaline protein- and chitin-degrading bacteria. Only amplicon sequence variants (ASVs) with relative abundance greater than 0.01% and detection rates above 60% were included to minimize false positives. The “WGCNA” package (version 1.6.9) was used to calculate network structures based on Spearman’s correlation coefficient with absolute values |*r*| > 0.6 and *p* < 0.05. In network analysis, nodes represent fundamental units, while edges indicate connections between nodes. Network topological properties, including average path length (APL), average clustering coefficient (ACC), and network density (ND), were computed using Gephi software (v0.9.2). APL represents the average distance between any two nodes within the network. Shorter path lengths indicate greater efficiency in nutrient cycling, energy flux, and signal transduction among species. ACC represents node aggregation closeness within the network, with higher values suggesting increased microbial sensitivity and rapid responsiveness to environmental disturbances. ND refers to the ratio of actual edges to maximum possible edges in the network. Higher ND indicates closer cooperative interactions among microorganisms.

## 3. Results

### 3.1. Changes in Straw Properties Under Different Decomposition Stages and N Application Rates

**Figure 2:**
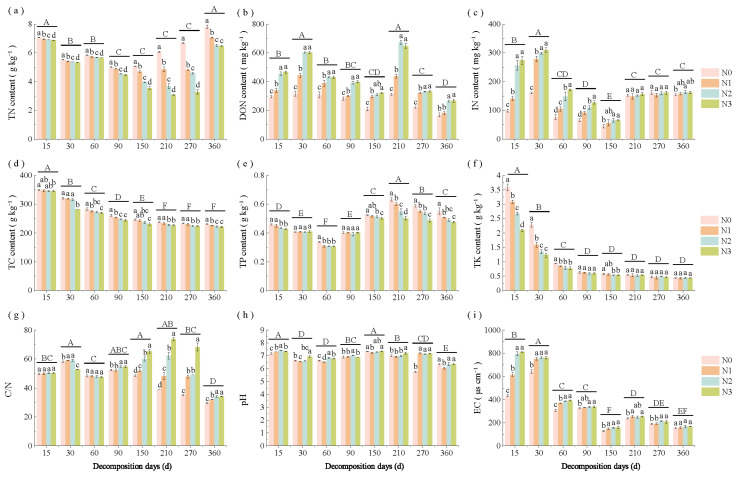
Changes in straw properties (**a**) TN, (**b**) DON, (**c**) IN, (**d**) TC, (**e**) TP, (**f**) TK, (**g**) C/N, (**h**) pH and (**i**) EC across decomposition stages and N application rates. Different lowercase letters above the bars indicate significant differences among N application rates within the same decomposition stage (*p* < 0.05), while different uppercase letters indicate significant differences among decomposition stages (*p* < 0.05). N0 (control, without N fertilizer), N1 (225 kg N/ha), N2 (300 kg N/ha), and N3 (375 kg N/ha); TN = total nitrogen; DON = dissolved organic nitrogen; IN = inorganic nitrogen; TC = total carbon; TP = total phosphorus; TK = total potassium; C/N = TC to TN ratio; EC = electrical conductivity.

Straw properties exhibited significant changes in response to N application rate and decomposition stage (Figure 2a–i). During decomposition, straw TN, IN, and TP contents showed an initial decrease, followed by an increase; TC, DON, and pH demonstrated continuous decline; TK and EC decreased rapidly; and the straw C/N ratio initially increased then decreased. At the equivalent decomposition stage, higher N application rates significantly (*p* < 0.05) reduced straw TN and increased DON levels. However, DON content showed no significant difference between N2 and N3 treatments. In addition, increased N rates significantly (*p* < 0.05) decreased straw TP content (days 150–360) and TK content (days 15–30), but increased IN level (days 15–90), C/N ratio (days 150–360), and EC (days 15–60).

### 3.2. Changes in Straw apr and chiA Gene Levels, α-Diversity, and Enzyme Activities Under Varying Decomposition Stages and N Application Rates

**Figure 3:**
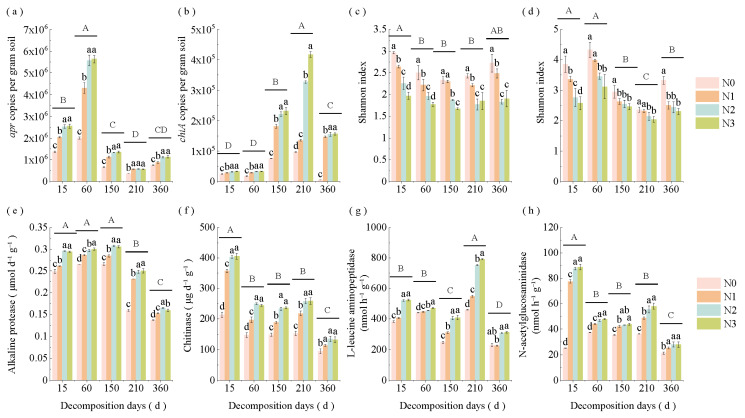
Changes in straw (**a**) *apr* and (**b**) *chiA* gene levels, α-diversity of alkaline protein- (**c**) and chitin-degrading (**d**) bacteria, and N hydrolase enzyme activity (**e**) alkaline protease, (**f**) chitinase, (**g**) L-leucine aminopeptidase and (**h**) N-acetylglucosaminidase across decomposition stages and N application rates. Different lowercase letters above the bars indicate significant differences among N application rates within the same decomposition stage (*p* < 0.05), while different uppercase letters indicate significant differences among decomposition stages (*p* < 0.05). N0 (control, without N fertilizer), N1 (225 kg N/ha), N2 (300 kg N/ha), and N3 (375 kg N/ha).

N application rates and decomposition stages significantly influenced straw *apr* and *chiA* gene levels, α-diversity, and N hydrolase activity (Figure 3a–h). The *apr* level increased during the early decomposition stage (days 15–60), followed by a gradual decline, while the *chiA* level showed an inverse pattern. The Shannon index of alkaline protein- and chitin-degrading bacteria initially decreased before increasing. In addition, the enzyme activities of straw AP, CAS, LAP, and NAG decreased over time, though with varying temporal patterns. AP activity significantly decreased (*p* < 0.05) on day 210, while LAP activity declined on day 150. CAS and NAG activities showed significant reduction on day 60 (Figure S1). Higher N application decreased (*p* < 0.05) the Shannon index of alkaline protein- and chitin-degrading bacteria but enhanced other indices. No significant differences were detected between N2 and N3 treatments.

### 3.3. Key Factors Regulating Straw N Hydrolase Enzymes

**Figure 4:**
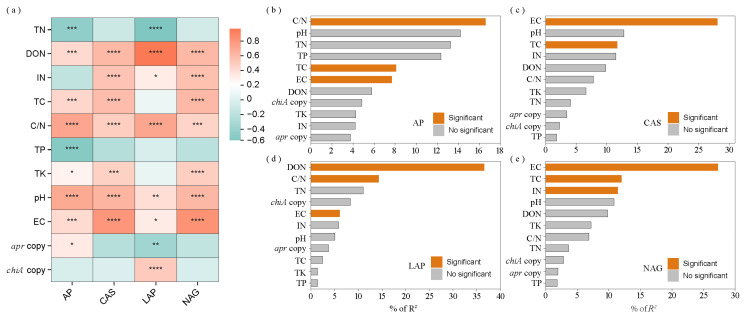
Heatmap and bar plots depicting (**a**) correlations between N hydrolase enzyme activities and straw biochemical parameters; (**b**) key determinants affecting AP activity, (**c**) CAS activity, (**d**) LAP activity, and (**e**) NAG activity. Green and red squares in the heatmap indicate negative and positive correlations, respectively. Asterisks *, **, *** and **** denote *p*  <  0.05, *p*  <  0.01, *p*  <  0.001 and *p*  <  0.0001, respectively. AP = alkaline protease; CAS = chitinase; LAP = L-leucine aminopeptidase; NAG = N-acetylglucosaminidase; TN = total nitrogen; DON = dissolved organic nitrogen; IN = inorganic nitrogen; TC = total carbon; TP = total phosphorus; TK = total potassium; C/N ratio = TC to TN ratio; EC = electrical conductivity.

Correlation analysis revealed that AP activity showed significant positive correlations with straw DON (r = 0.426), TC (r = 0.438), C/N ratio (r = 0.716), TK (r = 0.281), pH (r = 0.692), EC (r = 0.419), and *apr* copies (r = 0.325), and negative correlations with TN (r = −0.454) and TP (r = −0.515). CAS activity demonstrated significant positive correlations with DON (r = 0.608), IN (r = 0.546), TC (r = 0.582), C/N ratio (r = 0.488), TK (r = 0.468), pH (r = 0.629), and EC (r = 0.806). LAP activity exhibited significant positive correlations with straw DON (r = 0.967), IN (r = 0.318), C/N ratio (r = 0.712), pH (r = 0.396), EC (r = 0.321), and *chiA* copies (r = 0.321), and negative correlations with TN (r = −0.551) and *apr* copies (r = −0.356). NAG activity showed significant positive correlations with DON (r = 0.610), IN (r = 0.561), TC (r = 0.607), C/N (r = 0.456), TK (r = 0.486), pH (r = 0.614), and EC (r = 0.827) (Figure 4a).

Hierarchical partitioning analysis identified straw C/N ratio, TC, and EC as the primary factors significantly influencing AP activity, accounting for 16.59%, 8.09%, and 7.69% of the total variance, respectively (Figure 4b). Straw EC and TC were the major factors significantly affecting CAS activity, explaining 28.09% and 11.71% of the total variance, respectively (Figure 4c). Straw DON, C/N ratio, and EC were the principal factors significantly influencing LAP activity, accounting for 36.60%, 14.34%, and 6.07% of the total variance, respectively (Figure 4d). Similarly, straw EC, TC, and IN were the main factors significantly affecting NAG activity, explaining 27.27%, 12.00%, and 11.41% of the total variance, respectively (Figure 4e). The relationship between N hydrolase activity and DON was evaluated using the principal component (PC) scores derived from AP, CAS, LAP, and NAG activities. A strong linear regression relationship was observed between the PC1 score of N hydrolase enzyme activity and DON, as described by the equation: DON = 359.83 + 76.42 × PC1 (*R*^2^ = 0.66, *p* < 0.001) (Figure S2).

### 3.4. Changes in the Community Structures of Bacteria Involved in Straw Alkaline Protein and Chitin Degradation Under Different Decomposition Stages and N Application Rates

**Figure 5:**
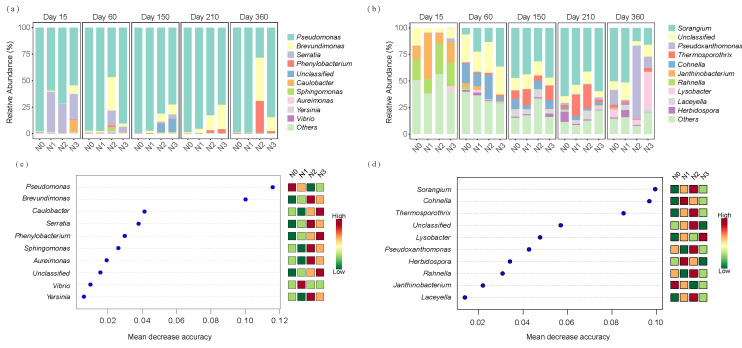
Relative abundances of the top 10 bacterial genera in (**a**) alkaline protein-degrading and (**b**) chitin-degrading communities. Top 10 biomarker genera for (**c**) alkaline protein-degrading and (**d**) chitin-degrading communities identified by random forest classification modeling. N0 represents the control without N fertilizer, N1 represents 225 kg N/ha, N2 represents 300 kg N/ha, and N3 represents 375 kg N/ha.

For alkaline protein-degrading bacteria, the genus *Pseudomonas* dominated with an average relative abundance of 81.41% (Figure 5a). During initial decomposition (day 15), *Serratia* ranked second (22.50%), but its abundance decreased rapidly over time. *Brevundimonas* and *Phenylobacterium* gradually increased, reaching peak abundances of 13.70% and 8.10%, respectively, on day 360. Random forest analysis and heatmap results indicated that *Pseudomonas* contributed most significantly to straw alkaline protein mineralization, followed by *Brevundimonas*. The relative abundance of *Pseudomonas* decreased with increasing N application, while *Brevundimonas* showed the opposite response (Figure 5c). The relative abundances of phyla in the alkaline protein-degrading bacterial community are shown in Figure S3a.

For chitin-degrading bacteria, the dominant genera on straw during early decomposition (day 15) included *Janthinobacterium* and *Rahnella*, with mean relative abundances of 21.05% and 21.36%, respectively (Figure 5b). However, *Janthinobacterium* and *Rahnella* showed rapid decline between days 60 and 210, while the relative abundance of *Sorangium* increased substantially, followed by *Cohnella* and *Thermosporothrix*, reaching their peak mean relative abundances on day 210 (38.96%), day 60 (8.09%), and day 210 (7.99%), respectively. The mean relative abundances of *Pseudoxanthomonas* and *Lysobacter* increased markedly, attaining 23.92% and 11.76%, respectively, by day 360. Random forest analysis and heatmap results indicated that during straw decomposition, *Sorangium* contributed the most to straw chitin mineralization, followed by *Cohnella* and *Thermosporothrix*. The relative abundances of these three genera initially increased and subsequently decreased with increasing N application (Figure 5d). The relative abundances of phyla in the chitin-degrading bacterial community are shown in Figure S3b.

**Figure 6:**
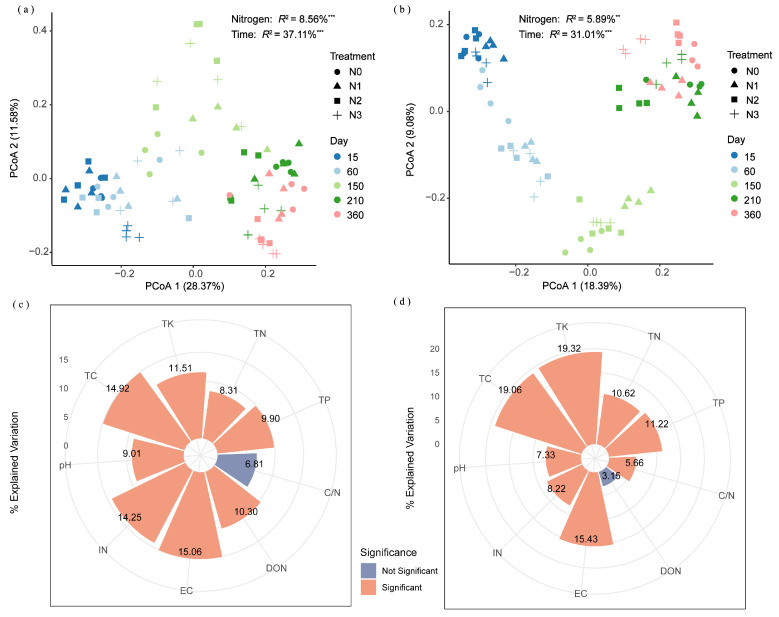
Principal coordinate analysis based on unweighted UniFrac distance demonstrating the effects of fertilizer treatments and decomposition duration on community structures of (**a**) alkaline protein-degrading and (**b**) chitin-degrading bacteria. Hierarchical partitioning analysis identified key factors influencing the community structures of (**c**) alkaline protein-degrading and (**d**) chitin-degrading bacteria. Asterisks ** and *** denote *p* < 0.01 and *p* < 0.001, respectively. N0 (control, without N fertilizer), N1 (225 kg N/ha), N2 (300 kg N/ha), and N3 (375 kg N/ha); TN = total nitrogen; DON = dissolved organic nitrogen; IN = inorganic nitrogen; TC = total carbon; TP = total phosphorus; TK = total potassium; C/N = TC to TN ratio; EC = electrical conductivity.

Unconstrained PCoA revealed that decomposition duration and N application significantly influenced the community succession of alkaline protein- and chitin-degrading bacteria. PERMANOVA showed that decomposition duration accounted for 37.11% (*p* < 0.001) and 31.01% (*p* < 0.001) of the community variance in alkaline protein- and chitin-degrading bacteria, respectively, while N application explained 8.56% (*p* < 0.001) and 5.89% (*p* < 0.01), respectively (Figure 6a,b). The PERMANOVA results demonstrated that decomposition duration was the primary driver shaping both bacterial communities, explaining approximately 4–5 times more variance than N fertilizer application management. Hierarchical partitioning analysis based on redundancy analysis identified EC, TC, IN, TK, DON, TP, pH and TN as the principal factors significantly influencing the community structure of alkaline protein-degrading bacteria, accounting for 15.06%, 14.92%, 14.25%, 11.51%, 10.30%, 9.90%, 9.01%, and 8.31% of the total variance, respectively (Figure 6c). TK, TC, EC, TP, TN, IN, pH, and the C/N ratio were the main factors significantly affecting variation in the community structure of chitin-degrading bacteria, explaining 19.32%, 19.06%, 15.43%, 11.22%, 10.62%, 8.22%, 7.33%, and 5.66% of the total variance, respectively (Figure 6d).

### 3.5. Co-Occurrence Network of Straw N-Mineralization Bacteria

**Figure 7:**
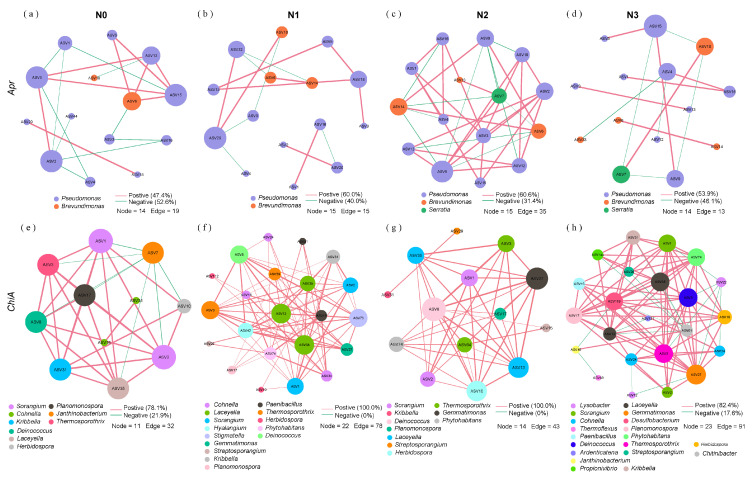
Co-occurrence networks of alkaline protein-degrading bacteria under (**a**) N0, (**b**) N1, (**c**) N2, and (**d**) N3 treatments, and chitin-degrading bacteria under (**e**) N0, (**f**) N1, (**g**) N2, and (**h**) N3 treatments. Red and green edges between nodes indicate positive and negative associations, respectively. The edge thickness between two nodes is proportional to Spearman’s correlation coefficients. Included connections represent strong (Spearman’s |r| > 0.6) and significant (*p* < 0.05) correlations. For N0, N1, N2, and N3 networks, the corresponding treatments (N0, N1, N2, and N3) from days 15, 60, 150, 210, and 360 were integrated into a combined dataset to construct each respective network. N0 (control, without N fertilizer), N1 (225 kg N/ha), N2 (300 kg N/ha), and N3 (375 kg N/ha).

Molecular ecological network analysis revealed distinct co-occurrence patterns of protein-degrading bacteria under varying N inputs during straw decomposition. The proportions of positive associations, ACC, and ND increased initially and then decreased with increasing N application, whereas APL showed the opposite pattern. N2 treatment exhibited the highest number of nodes, edges, positive associations, ACC, and ND, but the lowest number of negative associations and APL (Figure 7a–d; Table 2). For chitin-degrading bacteria, the proportions of positive associations, ACC, and ND increased and then decreased with increasing N application, while APL displayed the opposite trend. The N2 treatment demonstrated the highest number of positive associations, ACC, and ND, along with the lowest number of negative associations and APL (Figure 7e–h; Table 2).

### 3.6. PLS-PM Analysis

**Figure 8:**
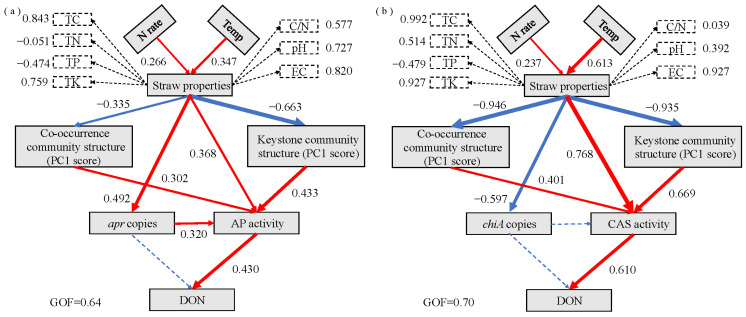
Pathway analysis of (**a**) AP and (**b**) CAS involved in the mineralization of straw residue N to DON. Rectangles represent observed or latent variables. Numbers adjacent to arrows indicate standardized path coefficients. The red and blue arrows indicate positive and negative effects, respectively. Dashed arrows represent non-significant effects. Numbers beside dashed rectangles indicate factor loadings of latent variables. Model goodness-of-fit (GoF) was evaluated using the GoF index. AP = alkaline protease; CAS = chitinase; TC = total carbon; TN = total nitrogen; TP = total phosphorus; TK = total potassium; DON = dissolved organic nitrogen; C/N ratio = TC to TN ratio; EC = electrical conductivity.

PLS-PM analysis revealed the relationships among environmental factors, straw properties, microbial community structure, and N hydrolase activity (Figure 8a,b). Using the random forest model, the top 15 ASVs with the highest importance scores for alkaline proteins and chitin mineralization were identified as keystone taxa (Tables S1 and S2). The analysis demonstrated that N application (0.226) and ground temperature (0.347) had significant positive effects on straw properties. Straw properties exhibited significant positive effects on *apr* copy numbers (0.492) and AP activity (0.368), while negatively affecting the co-occurring community (−0.335) and keystone taxa (−0.663) of alkaline protein-degrading bacteria. AP activity was directly influenced by straw properties, alkaline protein-degrading bacterial abundance, the co-occurring community (0.302), and keystone taxa (0.433). DON was significantly influenced solely by AP activity (0.430) (Figure 8a). These findings indicate three regulatory pathways through which N application and ground temperature influence AP-mediated depolymerization of straw TN to DON: (i) direct influence on AP activity through straw properties, (ii) modification of the co-occurring and keystone communities of alkaline protein-degrading bacteria, affecting AP activity, and (iii) changes in alkaline protein-degrading bacterial abundance, influencing AP activity. The results also demonstrated that ground temperature had a greater effect on straw properties than N application, while keystone taxa exhibited stronger influence on AP activity compared with the co-occurring community.

Regarding straw chitin-derived N mineralization, N application (0.237) and ground temperature (0.613) significantly and positively influenced the straw properties, which subsequently enhanced CAS activity (0.768), while negatively affecting *chiA* gene copy number (−0.597), the co-occurring community (−0.946), and keystone taxa (−0.935). CAS activity was directly influenced by straw properties, the co-occurring community (0.401), and keystone taxa (0.669), while DON was significantly affected only by CAS activity (0.610) (Figure 8b). The analysis revealed two regulatory pathways through which N application and ground temperature influence CAS-mediated depolymerization of straw TN to DON: (i) direct influence on CAS activity through straw properties and (ii) modification of the co-occurring and keystone communities, affecting CAS activity. Ground temperature demonstrated a greater impact on straw properties than N application, and keystone taxa showed stronger effects on CAS activity compared with the co-occurring community.

## 4. Discussion

### 4.1. Moderate N Input Enhances Microbial Cooperation and Enzyme Activities

N application accelerated straw N depolymerization, as evidenced by increased DON and IN levels, along with increased levels of N-depolymerizing genes (*apr* and *chiA*) and activities of key enzymes such as AP, CAS, LAP, and NAG (Figure 2a–c and Figure 4a). This enhancement results from the alleviation of microbial N limitation, stimulating population growth and promoting extracellular enzyme synthesis involved in straw decomposition [29,30,31]. However, a “marginal diminishing effect” emerged beyond the N2 rate (300 kg/ha), where additional N increases yielded minimal benefits (Figure 3e–g). This phenomenon may be attributed to multiple mechanisms: (1) increased EC under high-N conditions induces ion toxicity and osmotic stress, compromising microbial function; (2) inappropriate eco-enzymatic stoichiometry, where excess N alleviates N limitation but shifts constraints to other factors (e.g., C), limiting microbial metabolic efficiency [7,32,33]; and (3) the accumulation of metabolic end-products (e.g., NH_4_^+^, amino acids) potentially feedback-inhibiting enzyme production [34]. APL represents the average distance between network nodes; shorter APL indicates enhanced efficiency in material cycling, energy flow, and information transfer among species, enabling rapid disturbance responses. ACC measures node aggregation closeness, and higher ACC values indicate greater microbial sensitivity to environmental factors. ND represents the ratio of actual to possible network edges; higher ND values suggest stronger microbial cooperation [35,36,37]. Network analysis revealed that N2 treatment produced the most robust and resilient microbial community with complex, stable co-occurrence patterns for functional microbes, characterized by highest connectivity, positive associations, ACC, ND, and shortest APL (Figure 7a–h; Table 2). Optimized N input shapes microbial communities to enhance functional redundancy and facilitate resource exchange and stress response. Conversely, excessive N application (N3) resulted in fragmented networks with increased competitive interactions, suggesting reduced stability under high-N conditions. These findings demonstrate that optimal N management promotes synergistic microbial interactions while reinforcing soil ecosystem stability and adaptive capacity, thereby maintaining nutrient cycling functions.

### 4.2. Keystone Taxa Drive N Depolymerization at Different Decomposition Stages

The microbial community facilitating straw decomposition underwent dynamic succession, with distinct keystone taxa predominating at different decomposition stages and responding variably to N input. For alkaline protein depolymerization, *Pseudomonas* served as a fundamental genus throughout the process (Figure 5a,c), owing to its broad catabolic capacity, lignin-degrading ability, and tolerance to low temperature and salinity [38,39,40,41,42]. Its abundance, however, declined under higher N inputs, consistent with earlier reports [43]. In contrast, *Serratia* (early stage) and *Brevundimonas* (late stage) thrived with N addition [44]. *Serratia* grows rapidly under high N and degrades diverse organic compounds [45,46,47,48], while *Brevundimonas* adopts a K-strategy that supports survival in nutrient-limited late-stage environments through lignocellulose degradation [49,50,51,52].

Chitin degradation pathways proceeded through a distinctly specialized trophic succession that closely corresponded to changing substrate composition and thermal conditions. Early decomposition (15 days) was driven by psychrotolerant *Janthinobacterium* and *Rahnella*, which consumed labile carbon sources at low temperatures [41,53,54]. Mid-to-late stages (60–210 days) were dominated by thermophilic *Sorangium*, *Cohnella*, and *Thermosporothrix* (Figure 5b,d), which efficiently decomposed chitin and cellulose using heat-resistant enzymes. *Sorangium* displayed notable substrate versatility and antifungal activity [55,56]. The final stage (360 days) saw the rise of cryotolerant *Pseudoxanthomonas* and *Lysobacter*, with the latter increasing markedly under high N—likely due to its competitive traits, including antibacterial metabolites and hydrolase production [57,58,59,60,61]. These coordinated successional patterns represent sophisticated microbial adaptive responses to dynamically changing substrate composition, seasonal temperature fluctuations, and N availability gradients, illustrating how keystone taxa collectively influence microbial functional dynamics throughout the decomposition continuum.

### 4.3. Straw Properties and Temperature Are Primary Regulators of Enzyme-Mediated DON Production

PLS-PM elucidated the hierarchical control on straw N depolymerization, identifying two primary pathways controlling the conversion of straw N to DON. The first pathway demonstrated that straw properties, particularly TC and EC, directly influenced N hydrolase activities (Figure 8a,b). In saline soils, elevated EC directly inhibited enzyme conformation and function through ionic effects, while TC content determined the return on energy investment for microbial enzyme production. Eco-enzyme activities in saline soils were predominantly regulated by soil organic matter and EC, with these factors jointly controlling microbial activity within specific thresholds [62,63]. When 0.4 < EC < 2 dS/m, salinity indirectly suppressed microbial activity by reducing C input and accelerating C consumption. When EC exceeded 2 dS/m, soil microbial activity was predominantly governed by EC. Consequently, as EC increased, the primary determinant of enzyme activity shifted from soil organic matter to EC. This indicates that effective fertilization in coastal saline soils must consider both straw chemistry and salinity levels to prevent microbial function suppression. The second, more substantial pathway was indirect: straw properties primarily influenced the taxonomic and functional structure of the microbial community, which subsequently emerged as the primary determinant of enzyme production and DON levels (Figure 8a,b). This emphasizes that the composition and interactions of the microbial community, particularly the identity and function of keystone taxa, were more significant determinants of enzymatic efficiency than the level of functional genes. These findings correspond with Roy et al. [64], who demonstrated that microbial community composition, rather than species richness, influences decomposer functional diversity in soil. Community composition and interaction networks govern ecological functions, with core microbial taxa exerting greater influence than the overall community and significantly impacting ecosystem processes [22,65]. Beyond N management, temperature emerged as a more significant environmental regulator of straw decomposition than N application. Temperature influences the process through multiple mechanisms: direct control of microbial metabolic rates and enzyme reaction kinetics (Q_10_ effect), governance of straw substrate physicochemical properties, and regulation of microbial community succession. N hydrolase exhibited a Q10 value of approximately 1.59, indicating a 1.59-fold increase in reaction rate per 10 °C temperature rise [66]. The microbial taxa involved in straw N depolymerization in this study primarily exhibited psychrotolerant or thermotolerant characteristics. Thus, screening psychrotolerant and thermotolerant strains and constructing synthetic communities may significantly enhance straw N depolymerization efficiency.

## 5. Conclusions

This study demonstrates that N application rate and decomposition duration are critical factors regulating straw N mineralization in coastal saline paddy soil. A “marginal diminishing effect” occurred when N rate increased from 300 kg/ha to 375 kg/ha, beyond which N-depolymerizing functional gene levels and enzyme activities were inhibited. The application of 300 kg/ha N effectively mitigated microbial competition and enhanced mutualistic interactions, representing the optimal strategy for promoting straw N depolymerization. Keystone taxa, including *Pseudomonas*, *Brevundimonas*, *Sorangium*, *Cohnella*, and *Thermosporothrix*, were identified at different decomposition stages. Two main regulatory pathways were revealed: (i) straw properties (TC and EC) directly modulate enzyme activity; and (ii) straw properties indirectly affect enzyme activity and DON formation by shaping microbial community structure. Temperature exerted a stronger influence than N application, and keystone taxa demonstrated greater impact on enzymes than the overall community. These findings indicate that straw returning with application of 300 kg/ha N to coastal saline paddy soil accelerates straw decomposition while achieving reduced N fertilizer application. This fertilization strategy is important to achieve green development in coastal agriculture. Future research should focus on developing microbial inoculants based on these psychrotolerant and thermotolerant keystone strains to enhance straw N decomposition efficiency in saline soil environments.

## Figures and Tables

**Table 1:** Basic properties of tested soil and the nutrient and lignocellulose contents of tested straw.

**Soil Type**	**Organic C** **(g/kg)**	**TN** **(g/kg)**	**Alkaline N** **(mg/kg)**	**Available P** **(mg/kg)**	**Available K** **(mg/kg)**	**pH**	**EC** **(μs/cm)**	**Soil Salt Content** **(g/kg)**
Saline paddy soil	7.09	0.84	34.6	15.4	243.2	8.13	365.8	1.17
**Straw Type**	**TC** **(g/kg)**	**TN** **(g/kg)**	**TP** **(g/kg)**	**TK** **(g/kg)**	**Cellulose** **(%)**	**Hemicellulose** **(%)**	**Lignin** **(%)**	**pH**
Rice straw	384.3	8.78	0.87	12.3	36.7	24.0	13.4	6.83

Organic C = organic carbon; TN = total nitrogen; Alkaline N = alkaline nitrogen; Available P = available phosphorus; Available K = available potassium; TC = total carbon; TP = total phosphorus; TK = total potassium; EC = electrical conductivity.

**Table 2:** Topological properties of microbial interaction networks across treatments.

	Treatment	ACC	Change vs. N0 (%)	APL	Change vs. N0 (%)	ND	Change vs. N0 (%)
Alkaline protein-degrading bacteria	N0	0.42	-	1.99	-	0.29	-
	N1	0.43	2.38	1.79	−10.1	0.33	13.8
	N2	0.68	61.9	1.58	−20.6	0.49	69.0
	N3	0.50	19.1	2.26	13.5	0.28	−3.45
Chitin-degrading bacteria	N0	0.78	-	1.60	-	0.52	-
	N1	0.74	−5.13	1.61	0.63	0.48	−7.69
	N2	0.83	6.41	1.35	−15.6	0.68	30.8
	N3	0.74	−5.13	1.50	−6.25	0.54	3.85

N0 (control, without N fertilizer), N1 (225 kg N/ha), N2 (300 kg N/ha), and N3 (375 kg N/ha); ACC = average clustering coefficient; APL = average path length; ND = network density.

## Data Availability

The raw data supporting the conclusions of this article will be made available by the authors on request.

## Supplementary Materials

The following supporting information can be downloaded at: https://www.mdpi.com/article/10.3390/microorganisms13102333/s1, Figure S1: Heatmaps of straw N hydrolase activity across decomposition stages and N application rates; Figure S2: Regression of DON on PC1 score of N hydrolases; Figure S3. Relative abundance of the top five bacterial phyla in alkaline protein-degrading (a) and chitin-degrading (b) communities; Table S1: The top fifteen ASV of alkaline protein-degrading bacteria with the highest importance scores in the random forest model; Table S2: The top fifteen ASV of chitin-degrading bacteria with the highest importance scores in the random forest model.

## Abbreviations

The following abbreviations are used in this manuscript:

DON	Dissolved organic nitrogen
TC	Total carbon
TN	Total nitrogen
IN	Inorganic nitrogen
TP	Total phosphorus
TK	Total potassium
C/N	TC to TN ratio
EC	Electrical conductivity
AP	Alkaline protease
CAS	Chitinase
LAP	L-leucine aminopeptidase
NAG	N-acetylglucosaminidase
ACC	Average clustering coefficient
APL	Average path length
ND	Network density
