# Supplementary material for: Microbial Role in Straw Organic Matter Depolymerization to Dissolved Organic Nitrogen Under Nitrogen Fertilizer Reduction in Coastal Saline Paddy Soil
Source: Microorganisms. 2025 Oct 10;13(10):2333. doi: 10.3390/microorganisms13102333 (PMC12566125; doi:10.3390/microorganisms13102333)
Supplement: Supplementary file 1 [file microorganisms-13-02333-s001.zip › microorganisms-3833210-supplementary.pdf]

Supplementary materials

Manuscript Title: Microbial Role in Straw Organic Matter Depolymerization to Dissolved Organic Nitrogen Under Nitrogen Fertilizer Reduction in Coastal Saline Paddy Soil

The name(s) of the author(s): Xianglin Dai [1,2,3,*], Jianping Sun [1,2], Hao Li [1,2], Zijing Zhao [1,2], Ruiping Ma [1,2], Yahui Liu [1,2,*], Nan Shan [4], Yutao Yao [1,2] and Zhizhong Xue [1,2]

The affiliation(s) and address(es) of the author(s):

1 Institute of Coastal Agriculture, Hebei Academy of Agriculture and Forestry Sciences, Tangshan 063200, China; bhssjp@163.com (J.S.); nkybhslh@126.com (H.L.); zhaozijing94@163.com (Z.Z.); marp0825@126.com (R.M.); yutao890310@163.com (Y.Y.); nvtw_306675@sohu.com (Z.X.)
2 Tangshan Key Laboratory of Rice Breeding, Tangshan 063200, China
3 National Center of Technology Innovation for Comprehensive Utilization of Saline-Alkali Land, Dongying 257300, China
4 School of New Materials and Chemical Engineering, Tangshan University, Tangshan 063000, China; shannan@tsc.edu.cn

The e-mail address, telephone and fax numbers of the corresponding author: *Corresponding author: Tel.: +860315 8719028; Fax: +86 0315 8719028. E-mail: bhsdxl@126.com (X.D.), E-mail: bhslyh@126.com (Y.L.)

Bioinformatic analyses

Raw FASTQ files were demultiplexed using Trimmomatic (http://www.usadellab.org/cms/uploads/supplementary/Trimmomatic) and in-house Perl scripts based on sample-specific barcode sequences. The following criteria were applied: (i) the 300 bp reads were truncated at any position where the average quality score below 20 over a 10 bp sliding window, and truncated reads shorter than 50 bp were discarded; (ii) reads with exact barcode matches and up to two nucleotide mismatches in primer sequences were retained, while reads containing ambiguous bases were removed; (iii) only sequences with overlap regions longer than 10 bp were assembled based on their overlap. Reads that could not be assembled were discarded. Passed sequences were dereplicated and processed using the DADA2 algorithm (QIIME 2 recommended) to identify indel-mutations and nucleotide substitutions. Trimming and filtering were applied to paired reads, allowing a maximum of two expected errors per read (maxEE = 2). Following the merging of paired reads and chimera filtering, the phylogenetic affiliation of each *apr* and *chiA* gene sequence (herein called ASVs) was analyzed by blastx against the NR database with parameter "--evalue 1e-10 -k 10". After ASVs generation, the analysis of the data were operated by CFViSA plantform (http://www.cloud.biomicroclass.com/en/CFViSA), which contained microbiome analysis pipeline and nearly 80 analysis tools spanning simple sequence processing, visualization, and statistics available for the amplicon sequencing data.

Supplementary Figure S1

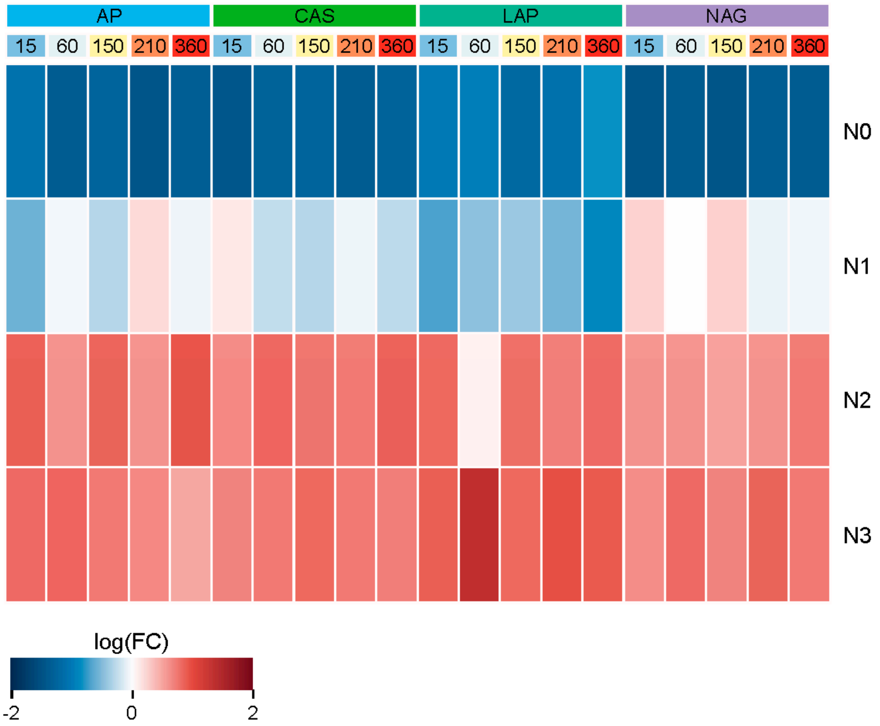

Figure S1. Heatmaps of straw N hydrolase activity across decomposition stages and N application rates. AP=alkaline protease; CAS=chitinase; LAP=L-leucine aminopeptidase; NAG=N-acetylglucosaminidase; N0 (control, without N fertilizer), N1 (225 kg N/ha), N2 (300 kg N/ha), and N3 (375 kg N/ha).

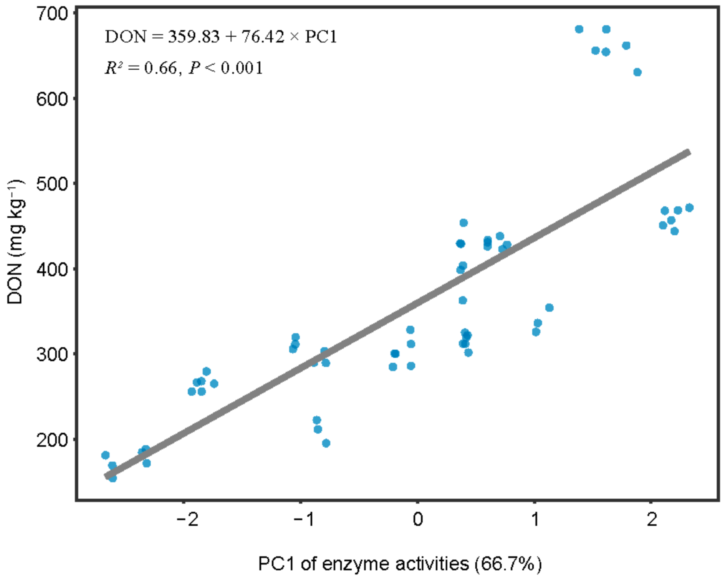

Figure S2. Regression of DON on PC1 score of N hydrolases.

Supplementary Figure S3

During straw decomposition, Pseudomonadota was the predominant phylum among the alkaline protein-degrading bacteria, with an average relative abundance of 98.89%. In contrast, the relative abundance of other phyla was lower, and their variation was negligible (Figure. S2a). For chitin-degrading bacteria, at the phylum level, the mean relative abundance of Pseudomonadota was highest on straw at day 15 (83.17%) but declined sharply before recovering to 39.62% by day 360. The mean relative abundance of Myxococcota increased rapidly to 40.0% between days 60 and 210, followed by a decrease to 32.99% by day 360. Bacillota and Chloroflexota exhibited a low-high-low trend throughout the straw decomposition period, reaching peak mean relative abundances of 22.47% at day 60, 15.94% at day 150, and 13.27% at day 210, respectively (Figure. S2b).

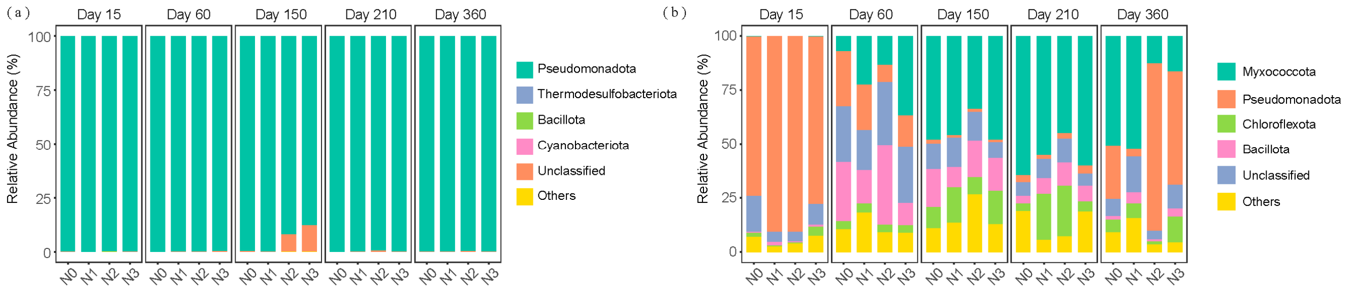

Figure S3. Relative abundance of the top five bacterial phyla in alkaline protein-degrading (a) and chitin-degrading (b) communities.

Table S1 The top fifteen ASV of alkaline protein-degrading bacteria with the highest importance scores in the random forest model

	ASV id	Taxonomy						
		Domain	Phylum	Class	Order	Family	Genus	Species
Alkaline protein-degrading bacteria	ASV6	Bacteria	Pseudomonadota	Alphaproteobacteria	Caulobacterales	Caulobacteraceae	*Brevundimonas*	*Brevundimonas sp.*
	ASV4	Bacteria	Pseudomonadota	Gammaproteobacteria	Pseudomonadales	Pseudomonadaceae	*Pseudomonas*	*Pseudomonas veronii*
	ASV7	Bacteria	Pseudomonadota	Gammaproteobacteria	Enterobacterales	Yersiniaceae	*Serratia*	
	ASV3	Bacteria	Pseudomonadota	Gammaproteobacteria	Pseudomonadales	Pseudomonadaceae	*Pseudomonas*	*Pseudomonas veronii*
	ASV2	Bacteria	Pseudomonadota	Gammaproteobacteria	Pseudomonadales	Pseudomonadaceae	*Pseudomonas*	*Pseudomonas veronii*
	ASV14	Bacteria	Pseudomonadota	Alphaproteobacteria	Caulobacterales	Caulobacteraceae	*Brevundimonas*	*Brevundimonas sp.*
	ASV5	Bacteria	Pseudomonadota	Gammaproteobacteria	Pseudomonadales	Pseudomonadaceae	*Pseudomonas*	*Pseudomonas veronii*
	ASV19	Bacteria	Pseudomonadota	Alphaproteobacteria	Caulobacterales	Caulobacteraceae	*Brevundimonas*	*Brevundimonas sp.*
	ASV20	Bacteria	Pseudomonadota	Gammaproteobacteria	Pseudomonadales	Pseudomonadaceae	*Pseudomonas*	
	ASV62	Bacteria	Pseudomonadota	Gammaproteobacteria	Pseudomonadales	Pseudomonadaceae	*Pseudomonas*	*Pseudomonas veronii*
	ASV1	Bacteria	Pseudomonadota	Gammaproteobacteria	Pseudomonadales	Pseudomonadaceae	*Pseudomonas*	*Pseudomonas veronii*
	ASV9	Bacteria	Pseudomonadota	Gammaproteobacteria	Pseudomonadales	Pseudomonadaceae	*Pseudomonas*	*Pseudomonas fluorescens*
	ASV12	Bacteria	Pseudomonadota	Gammaproteobacteria	Pseudomonadales	Pseudomonadaceae	*Pseudomonas*	*Pseudomonas veronii*
	ASV16	Bacteria	Pseudomonadota	Gammaproteobacteria	Pseudomonadales	Pseudomonadaceae	*Pseudomonas*	*Pseudomonas veroni*
	ASV33	Bacteria	Pseudomonadota	Alphaproteobacteria	Caulobacterales	Caulobacteraceae	*Brevundimonas*	*Brevundimonas denitrificans*

Table S2 The top fifteen ASV of chitin-degrading bacteria with the highest importance scores in the random forest model

	ASV id	Taxonomy						
		Domain	Phylum	Class	Order	Family	Genus	Species
Chitin-degrading bacteria	ASV119	Bacteria	Thermodesulfobacteriota	Desulfobacteria	Desulfobacterales	Desulfobacteriaceae	*Desulfobacterium*	*uncultured Desulfobacterium sp.*
	ASV1	Bacteria	Myxococcota	norank	Polyangiales	Polyangiaceae	*Sorangium*	*Sorangium cellulosum*
	ASV64	Bacteria	Chloroflexota	Ktedonobacteria	Ktedonobacterales	Thermosporotrichaceae	*Thermosporothrix*	*Thermosporothrix hazakensis*
	ASV264	Bacteria	Actinomycetota	Actinomycetes	Streptosporangiales	Streptosporangiaceae	*Herbidospora*	*Herbidospora cretacea*
	ASV2	Bacteria	Myxococcota	norank	Polyangiales	Polyangiaceae	*Sorangium*	*Sorangium cellulosum*
	ASV163	Bacteria	Bacillota	Bacilli	Bacillales	Thermoactinomycetaceae	*Laceyella*	*Laceyella sacchari*
	ASV42	Bacteria	Myxococcota	Myxococcia	Myxococcales	Archangiaceae	*Hyalangium*	*Hyalangium versicolor*
	ASV35	Bacteria	Bacillota	Bacilli	Bacillales	Thermoactinomycetaceae	*Laceyella*	*Laceyella sacchari*
	ASV164	Bacteria	Bacillota	Bacilli	Bacillales	Thermoactinomycetaceae	*Laceyella*	*Laceyella sacchari*
	ASV38	Bacteria	Bacillota	Bacilli	Bacillales	Thermoactinomycetaceae	*Laceyella*	*Laceyella sacchari*
	ASV126	Bacteria	Pseudomonadota	Gammaproteobacteria	Enterobacterales	Enterobacteriaceae	*Enterobacter*	*Enterobacter asburiae*
	ASV40	Bacteria	Pseudomonadota	Betaproteobacteria	Burkholderiales	Oxalobacteraceae	*Janthinobacterium*	*Janthinobacterium sp. 67*
	ASV21	Unclassified						
	ASV31	Bacteria	Actinomycetota	Actinomycetes	Propionibacteriales	Kribbellaceae	*Kribbella*	*Kribbella sp. ALI-6-A*
	ASV3	Bacteria	Chloroflexota	Ktedonobacteria	Ktedonobacterales	Thermosporotrichaceae	*Thermosporothrix*	*Thermosporothrix hazakensis*
